# Associations of sleep apnea risk and oxygen desaturation indices with cerebral small vessel disease burden in patients with stroke

**DOI:** 10.3389/fneur.2022.956208

**Published:** 2022-08-25

**Authors:** Xiaodi Liu, David Chi-Leung Lam, Henry Ka-Fung Mak, Mary Sau-Man Ip, Kui Kai Lau

**Affiliations:** ^1^Division of Neurology, Department of Medicine, Li Ka Shing Faculty of Medicine, The University of Hong Kong, Pokfulam, Hong Kong SAR, China; ^2^Division of Respiratory Medicine, Department of Medicine, Li Ka Shing Faculty of Medicine, The University of Hong Kong, Pokfulam, Hong Kong SAR, China; ^3^Department of Diagnostic Radiology, Li Ka Shing Faculty of Medicine, The University of Hong Kong, Pokfulam, Hong Kong SAR, China; ^4^The State Key Laboratory of Brain and Cognitive Sciences, The University of Hong Kong, Pokfulam, Hong Kong SAR, China

**Keywords:** obstructive sleep apnea, oxygen desaturation, nocturnal hypoxemia, small vessel disease, stroke, transient ischemic attack

## Abstract

**Background:**

Obstructive sleep apnea (OSA) is associated with cerebral small vessel disease (CSVD). Nonetheless, whether OSA-risk determined by a simple screening questionnaire or indices quantifying nocturnal hypoxemia other than the conventional apnea–hypopnea index (AHI) by the home sleep apnea test (HSAT) associated with CSVD burden remains uncertain.

**Methods:**

From 2018 to 2021, we recruited patients with transient ischemic attack (TIA)/minor stroke from the Queen Mary Hospital Acute Stroke Unit and TIA/Stroke Outpatient Clinics. Logistic regression models were applied to determine the association of baseline OSA-risk (on STOP-BANG questionnaire) or HSAT-derived indices quantifying nocturnal hypoxemia with global burden/individual markers of CSVD on MRI. Indices included oxygen desaturation (≥3%) index (ODI), minimum oxygen saturation (SpO_2_), percentage of total sleep time with an oxygen saturation <90% (CT90%), and desaturation duration (≥3%, DesDur).

**Results:**

In 283 patients with TIA/minor stroke (mean age 65 years, 64% men), OSA-risk was significantly associated with total CSVD score (multivariate-adjusted odds ratio: 1.23, 95% confidence interval 1.01–1.51), presence of lacunes [1.39 (1.09–1.79)] and burden of basal ganglia PVSs [1.32 (1.06–1.67)]. In 85/283 patients who completed HSAT, neither AHI, minimum SpO_2_ nor CT90% was associated with CSVD burden. Nonetheless, ODI and DesDur remained significantly associated with total CSVD score after covariate adjustment: ODI [1.04 (1.01–1.07)] and DesDur [1.04 (1.01–1.08)].

**Conclusion:**

In patients with TIA/minor stroke, high OSA-risk was associated with a greater CSVD burden. Oxygen desaturation indices (ODI and DesDur) rather than AHI were independently associated with global CSVD burden, indicating that longer and more severe desaturations may contribute to the pathogenesis of CSVD.

## Introduction

Obstructive sleep apnea (OSA) is a common and modifiable risk factor for stroke (1, 2). Left undiagnosed and untreated, stroke patients with OSA may be at increased risk of impaired functional and cognitive capacity (3), and may have a higher risk of recurrent stroke and death (4). Recent meta-analyses have shown that moderate-to-severe OSA is positively associated with cerebral small vessel disease (CSVD) (5, 6), a chronic vasculopathy that accounts for up to 20% of all strokes (7). Screening for OSA after stroke using standard diagnostic tests, such as polysomnography (PSG) or home sleep apnea test (HSAT), is recommended (8, 9), but access to these tests is often limited. Questionnaires have also been developed and validated to identify patients at high risk, among which the 8-item STOP-BANG questionnaire appears to have the highest sensitivity within the stroke population (10, 11).

Several studies have shown that a high OSA-risk is associated with an increased risk of intracerebral hemorrhage (12), impaired cerebrovascular reactivity (13), and presence of intracranial carotid artery calcification (14) after stroke. OSA and CSVD may share similar pathophysiological mechanisms *via* common vascular risk factors, such as hypertension. Nonetheless, it is not known whether OSA-risk is associated with the burden of CSVD in patients with transient ischemic attack (TIA)/minor stroke.

The apnea–hypopnea index (AHI) is the most widely used index to diagnose and define OSA severity using overnight PSG and HSAT. But it has recently been shown to have limited capacity to predict adverse clinical outcomes (15) or response to nasal continuous positive airway pressure (nCPAP) treatment in patients with OSA (16). In several large prospective cohort studies, a number of indices [e.g., hypoxic burden, desaturation duration, and percentage of total sleep time with SpO_2_ <90% (CT90%)] quantifying the depth and duration of oxygen desaturation were shown to have better prognostic value than AHI for outcomes, such as incident major adverse cardiovascular events (17), heart failure (18), incident stroke (19), and mortality (20). Yet, whether these indices are associated with magnetic resonance imaging (MRI) markers of CSVD has not been explored.

We aimed to determine whether OSA-risk estimated using STOP-BANG and oxygen desaturation indices identified using HSAT were independently associated with CSVD burden in patients with TIA/minor stroke.

## Materials and methods

### Design and setting

We prospectively recruited predominantly Chinese patients with a new diagnosis of TIA/minor stroke [National Institute of Health Stroke Scale (NIHSS) score <7] from the Acute Stroke Unit and TIA/Minor Stroke Clinic of Queen Mary Hospital, Hong Kong, from 2018–2021. Inclusion criteria were age ≥18 years, availability of a brain MRI and completed STOP-BANG questionnaire (21) at baseline (within 1 month of symptom onset). Clinical and demographic variables were recorded at baseline along with information about vascular risk factors (hypertension, hyperlipidemia, diabetes mellitus, history of stroke, atrial fibrillation, smoking, alcohol use), and type and etiology of stroke using the Trial of ORG 10172 in Acute Stroke Treatment (TOAST) criteria (22). Definition of vascular risk factors is listed in Supplementary Methods.

### Neuroimaging acquisition and analyses

All participants underwent a brain MRI at baseline according to protocols that have been described previously (23). One rater (XDL) who was trained by a consultant neuroradiologist (HKFM) coded all MRI scans for the presence and burden of white matter hyperintensities (WMH, periventricular and deep), lacunes, basal ganglia and centrum semiovale perivascular spaces (BG- and CSO-PVSs), cerebral microbleeds, as well as the presence of recent subcortical infarct, according to the STRIVE guidelines (24) and validated rating scales (25–27). The global burden of CSVD was calculated using the total CSVD score (23). Brain atrophy was assessed in the deep (ventricular enlargement) and superficial (gyral enlargement) regions against a validated normal aging reference template from the lowest (1) to the highest atrophy quantile (6, 28). The degree of medial temporal lobe atrophy (MTA) was assessed using the MTA scale (29). Ten random cases were cross-checked for each CSVD marker with excellent intra-rater reliability (Cohen's kappa 0.75–1).

### Overnight HSAT sub-study

We invited all eligible subjects to compete in an overnight home sleep study within 1 year after TIA/stroke onset using a validated device (NOX-T3, Nox Medical Inc. Reykjavik, Iceland) (30) unless they already had a known history of sleep apnea diagnosed by overnight PSG or HSAT. Details of the manual scoring methods are listed in Supplementary Methods. OSA severity was categorized as none or mild (AHI <15/h) or moderate to severe (AHI ≥ 15/h). In addition to AHI, ODI, minimum SpO_2_, and CT90% were recorded. A novel index, desaturation duration (DesDur), that has been recently described (31, 32) was also extracted. ODI was calculated as the average events during which oxygen saturation decreased by ≥3% from baseline. DesDur was calculated as the total time of oxygen saturation decrease ≥3% from baseline divided by the total sleep time. CT90% was calculated as the proportion of cumulative sleep time with SpO_2_ below 90% during total sleep time.

### Statistical analysis

We evaluated differences between a low (0–2) and an intermediate–high (>2) OSA-risk group stratified by STOP-BANG score, using independent *t*-tests for continuous normal measures and the Mann–Witney U tests, or Chi-square tests as appropriate.

The correlation of OSA-risk with CSVD markers was evaluated by Spearman's rank correlation, and the association of OSA-risk with CSVD markers was further investigated using ordinal logistic regression in: (1) an unadjusted model; (2) a model adjusted for age and sex; and (3) a multivariate model with additional adjustment of TIA/stroke history, vascular risk factors (baseline blood pressure, hyperlipidemia, diabetes, atrial fibrillation, smoking), and alcohol use, all potential confounders associated with CSVD burden. We did not adjust for BMI as it is already included in the total STOP-BANG score (one score assigned in individuals with BMI > 30 kg/m^2^).

In the HSAT sub-study, the diagnostic performance of the STOP-BANG score was assessed against AHI and similarly examined the correlations and associations between AHI and oxygen desaturation indices with global CSVD burden using ordinal logistic regression models.

All statistical tests were two-sided with a *p-*value <0.05 considered statistically significant. All statistical analyses were performed using R (version 4.1.0, R Foundation for Statistical Computing, Vienna, Austria).

### Standard protocol approvals and participant consent

This study was approved by the Institutional Review Board of the University of Hong Kong/Hospital Authority Hong Kong West Cluster (UW18-361). All study participants provided written informed consent at recruitment. This study was performed and reported following the Strengthening the Reporting of Observational Studies in Epidemiology guidelines (33).

## Results

**Figure 1:**
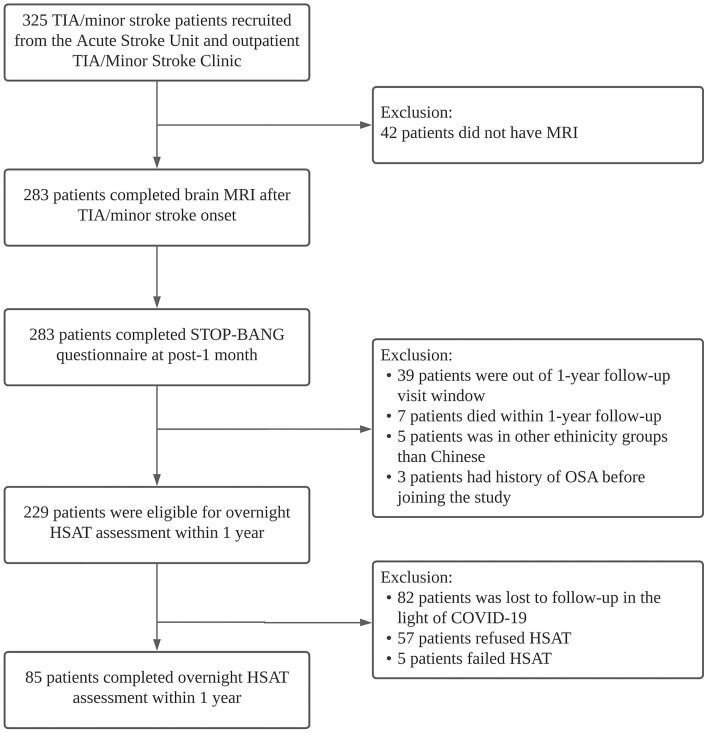
Study flow diagram. MRI, magnetic resonance imaging; TIA, transient ischemic attack; HSAT, home sleep apnea test.

Of the 325 patients recruited, 283 completed all baseline assessments and were included in the analysis (Figure 1). Brain MRI was performed at a median of 5 days (interquartile range, IQR three–eight days) after stroke onset. The mean age of the study population was 65.2 ± 12.0 years, and 64% were men. The mean baseline NIHSS score was 1.5 ± 1.6 and the mean body mass index (BMI) 24.2 ± 3.6. Minor stroke (98.1% ischemic) was diagnosed in 165 patients (58.3%) and TIA in 118 (41.7%).

Based on STOP-BANG, 170/283 patients (60.1%) were identified as being at intermediate–high OSA-risk. Compared with patients with low OSA-risk, those with intermediate–high OSA-risk were more likely to be men, with a higher prevalence of hypertension (and hence higher blood pressure), and were more likely to be smokers and alcohol drinkers. They were also more likely to have diffusion-weighted imaging (DWI)-positive lesions, higher CSVD burden, and more severe cerebral atrophy (all *p* < 0.05) (Table 1). There were otherwise no significant differences in age or proportion of patients with diabetes, hyperlipidemia, atrial fibrillation, and stroke history between the two groups (all *p* > 0.05). The subtypes, etiology, and severity of stroke also did not differ between the two groups (all *p* > 0.05).

**Table 1:** Baseline clinical and imaging characteristics of the study population.

	**All, *n* = 283**	**Low OSA-risk, *n* = 113**	**Intermediate–high OSA-risk, *n* = 170**	***p-*value**
**Clinical characteristics**				
Age, years	65.2 ± 12.0	63.9 ± 13.4	66.1 ± 11.0	0.215
Male, %	180 (63.6)	43 (38.1)	137 (79.6)	**<0.001**
BMI, kg/m^2^	24.2 ± 3.6	23.1 ± 3.2	24.9 ± 3.7	**<0.001**
Hypertension, %	158 (55.8)	46 (40.7)	112 (65.9)	**<0.001**
Hyperlipidemia, %	97 (34.3)	39 (34.5)	58 (34.1)	1
Diabetes, %	81 (28.6)	32 (28.3)	49 (28.8)	1
Atrial fibrillation, %	32 (11.3)	11 (9.7)	21 (12.4)	0.624
Prior TIA/stroke, %	35 (12.4)	12 (10.6)	23 (13.5)	0.587
Ever-smokers, %	84 (29.7)	19 (16.8)	65 (38.2)	**<0.001**
Alcohol drinker, %	59 (20.8)	16 (14.2)	43 (25.3)	**0.035**
Systolic blood pressure, mmHg	136 ± 18	133 ± 18	137 ± 18	0.054
Diastolic blood pressure, mmHg	77 ± 13	75 ± 12	78 ± 13	**0.019**
**Stroke characteristics**				
Stroke subtype, %				0.237
TIA	118 (41.7)	54 (47.8)	64 (37.6)	
Minor ischemic stroke	162 (57.2)	58 (51.3)	104 (61.2)	
Minor hemorrhagic stroke	3 (1.1)	1 (0.9)	2 (1.2)	
TOAST classification, %				0.123
Small vessel occlusion	86 (30.4)	28 (24.8)	58 (34.1)	
Other etiological subtypes	197 (69.6)	85 (74.2)	112 (65.9)	
NIHSS score	1.5 ± 1.6	1.5 ± 1.6	1.5 ± 1.6	0.767
**Imaging characteristics**				
Number with DWI positive lesion, %	176 (62.2)	59 (52.2)	117 (68.8)	**0.007**
Total CSVD score				**<0.001**
0	82 (29.0)	46 (40.7)	36 (21.2)	
1	74 (26.1)	28 (24.8)	46 (27.1)	
2	52 (18.4)	22 (19.5)	30 (17.6)	
3	47 (16.6)	10 (8.8)	37 (21.8)	
4	28 (8.9)	7 (6.2)	21 (12.4)	
Lacunes, %	114 (40.3)	32 (28.3)	82 (48.2)	**0.001**
Cerebral microbleeds^*^, %				
Presence	59 (20.8)	17 (15.0)	42 (24.7)	**0.047**
≥5	24 (8.5)	6 (5.3)	18 (10.7)	0.287
Moderate-extensive WMH, %	90 (31.8)	26 (23.0)	64 (37.6)	**0.014**
Basal ganglia PVS, %				**<0.001**
<10	133 (47.0)	68 (60.2)	65 (38.2)	
10–20	115 (40.6)	40 (35.4)	75 (44.1)	
>20	35 (12.4)	5 (4.4)	30 (17.6)	
Central semiovale PVS, %				**0.019**
<10	112 (39.6)	56 (49.6)	56 (32.9)	
10–20	126 (44.5)	43 (38.1)	83 (48.8)	
>20	45 (15.9)	14 (12.4)	31 (18.2)	
Total brain atrophy score, %				**0.049**
0–4	140 (49.5)	66 (58.4)	74 (43.5)	
5–8	93 (32.9)	30 (26.5)	63 (37.1)	
9–12	50 (17.7)	17 (15.0)	33 (19.4)	
MTA score	2.2 ± 1.0	2.0 ± 1.0	2.3 ± 1.0	**0.003**

Data are presented as mean ± standard deviation or number (percentage).

BMI, body mass index; DWI, diffusion-weighted imaging; PVS, perivascular space; MTA, medial temporal lobe atrophy; OSA, obstructive sleep apnea; CSVD, cerebral small vessel disease; TIA, transient ischemic attack; TOAST, Trial of ORG 10172 in Acute Stroke Treatment; WMH, white matter hyperintensity.

* Missing data for one subject.

Moderate-extensive WMH: periventricular WMH Fazekas score three or deep WMH Fazekas score 2–3; Total brain atrophy score, deep atrophy score + superficial atrophy score, ranging from 0 to 12.

Bold values indicate statistical significance with p value < 0.05.

OSA-risk determined by total STOP-BANG score was correlated with all MRI markers of CSVD (all *p* < 0.05) except for cerebral microbleeds and periventricular WMH (Supplementary Table 1). The positive association of OSA-risk with global CSVD burden remained significant after adjusting for age, sex, vascular risk factors, and alcohol use [multivariate-adjusted odds ratio (OR): 1.23, 95% confidence interval (CI) 1.01–1.51]. Compared with patients with low OSA-risk (STOP-BANG score 0–2), those at intermediate–high OSA-risk (STOP-BANG>2) tended to have worse CSVD burden [multivariate-adjusted OR 1.89 (1.16, 3.12)]. Higher OSA-risk was also significantly associated with the presence of lacunes [multivariate-adjusted OR 1.39 (1.09–1.79)] and burden of BG-PVSs [1.32 (1.06–1.67)] (Table 2). Significant associations of OSA-risk with WMH, CSO-PVSs and cerebral atrophy scores were also noted in univariate analysis and were attenuated after adjusting for age and sex. No association of OSA-risk with the presence of cerebral microbleeds was identified (Table 2).

**Table 2:** Association of OSA-risk with MRI markers of CSVD (*n* = 283).

**OSA-risk**	**CSVD markers**	**Outcome variables**	**Unadjusted OR (95% CI)**	**Age and sex adjusted OR (95% CI)**	Multivariate-adjusted^*^OR (95% CI)
Intermediate–high OSA-risk (STOP-BANG score >2)	Global CSVD burden	Total CSVD score	**1.41 [1.19–1.69]**	**1.25 [1.03–1.52]**	**1.89 [1.16–3.12]**
Total STOP-BANG score	Global CSVD burden	Total CSVD score	**2.39 [1.55–3.71]**	**2.02 [1.25–3.28]**	**1.23 [1.01–1.51]**
	Lacune	Presence of lacunes	**1.47 [1.20–1.82]**	**1.39 [1.10–1.77]**	**1.39 [1.09–1.79]**
	Microbleeds	Presence of microbleeds	1.16 [0.92–1.46]	1.08 [0.82–1.41]	1.15 [0.85–1.54]
	WMH	Periventricular Fazekas score	**1.20 [1.00–1.45]**	1.13 [0.90–1.41]	1.10 [0.87–1.39]
		Deep Fazekas score	**1.24 [1.04–1.49]**	1.09 [0.89–1.35]	1.06 [0.86–1.32]
	PVS	Basal ganglia PVS score	**1.47 [1.23–1.78]**	**1.33 [1.07–1.66]**	**1.32 [1.06–1.67]**
		Central semiovale PVS score	**1.36 [1.13–1.64]**	1.21 [0.99–1.50]	1.20 [0.97–1.48]
	Brain atrophy	Total brain atrophy score quantile	**1.24 [1.03–1.49]**	0.98 [0.78–1.23]	0.98 [0.77–1.25]
		MTA score	**1.22 [1.02–1.46]**	0.96 [0.78–1.18]	0.93 [0.75–1.16]

CI, confidence interval; MTA, medial temporal lobe atrophy; OR, odds ratio; CSVD, cerebral small vessel disease; PVS, perivascular space; WMH, white matter hyperintensities.

* Adjusted for age, sex, history of TIA/stroke, vascular risk factors (baseline blood pressure, hyperlipidemia, diabetes, atrial fibrillation, smoking), and alcohol use.

Bold values indicate statistical significance with p value < 0.05.

Of the 229 eligible subjects, 85 (mean age 62.7 ± 11.0 years, 66% men) underwent overnight HSAT a median of 13.4 months (IQR 12.3–14.3) after TIA/stroke onset (Figure 1). Patients who did complete HSAT were older (*p* = 0.038). There were otherwise no significant differences in the demographics or vascular risk factors among patients who underwent HSAT compared with those who did not (Supplementary Table 2). STOP-BANG (area under ROC curve: 0.626–0.701) showed acceptable diagnostic performance compared with AHI (Supplementary Figure 1). The mean AHI was 17.8 ± 14.4, and the mean ODI was 16.7 ± 13.9. The median value of DesDur, CT90%, and minimum SpO_2_ was 12.2% (IQR 8.3–21.5%), 1.7% (0.4–4.6%), and 83.0% (79.0–86.0%), respectively. Using HSAT, 41 patients (48.2%) were classified as having moderate-severe OSA.

The correlation was stronger for ODI, DesDur, and CT90% with total CSVD score than for AHI or minimum SpO_2_ (Supplementary Table 3). The associations of ODI and DesDur with CSVD burden remained significant after adjusting for confounding factors: ODI [1.04 (1.01–1.07), *p* = 0.036], DesDur [1.04 (1.01–1.08), *p* = 0.036]. However, no significant associations between AHI and total CSVD score were noted after adjustment of confounding factors [1.03 (1.00–1.06), *p* = 0.056] (Table 3). AHI, ODI, and DesDur were also significantly associated with burden of BG-PVSs: AHI [1.04 (1.01–1.07)], ODI: [1.04 (1.01–1.08)], DesDur: [1.05 (1.01–1.10)] (all *p* < 0.05). Only ODI remained significantly associated with deep WMH burden after covariates adjustment: [1.04 (1.01–1.08), *p* = 0.049] (Supplementary Tables 4, 5).

**Table 3:** Association of AHI and oxygen desaturation indices with total CSVD score (*n* = 85).

**Indices**	**Unadjusted OR (95% CI)**	Multivariate-adjusted^*^OR (95% CI)
AHI	1.03 [1.00–1.06]	1.03 [1.00–1.06]
ODI	**1.03 [1.00–1.06]**	**1.04 [1.01–1.07]**
DesDur	**1.04 [1.00–1.08]**	**1.04 [1.01–1.08]**
Minimum SpO_2_, %	0.99 [0.93–1.05]	1.00 [0.93–1.07]
CT90%	1.04 [1.00–1.09]	1.02 [0.97–1.07]

Bold values indicate statistical significance with p-value < 0.05.

AHI, apnea–hypopnea index; CI, confidence interval; CT90%, percentage of total sleep time with an oxygen saturation <90%; DesDur, percentage of total desaturation time from total sleep time; CSVD, cerebral small vessel disease; ODI, oxygen desaturation index; OR, odds ratio.

* Adjusted for age, sex, vascular risk factors (hypertension, diabetes, atrial fibrillation, history of stroke/TIA, BMI, smoking), alcohol use, and total sleep time.

## Discussion

In this study, we demonstrated that TIA/minor stroke patients with higher OSA-risk as determined by the STOP-BANG questionnaire had a greater CSVD burden. We also found that hypoxic burden, as determined by ODI and DesDur, was more significantly associated with global CSVD burden than AHI. Our findings indicate that features of nocturnal hypoxemia, such as the duration of intermittent desaturation, may provide additional information about OSA severity and may be important in the pathogenesis of CSVD.

In our main study, we used STOP-BANG to estimate the risk of OSA, with around 60% of participants categorized as intermediate–high OSA-risk. This feature is consistent with recent meta-analyses that have demonstrated a high prevalence of OSA among patients with stroke (2, 34, 35). In our HSAT sub-study, STOP-BANG also showed acceptable diagnostic performance against HSAT. These results are also in-line with a recent meta-analysis in which of intermediate–high OSA-risk detected using STOP-BANG had excellent sensitivity (>90%) against PSG testing (36).

Several large cohort studies in patients with OSA have explored the association of novel desaturation metrics with clinical outcomes. For instance, the SAVE trial showed that desaturation duration and desaturation/resaturation time ratio were predictive of future risk of heart failure (18). In the Osteoporotic Fractures in Men Sleep Study, nocturnal hypoxia measured by CT90% was independently associated with subsequent risk of stroke (19) and cardiovascular mortality (37). Our previous sleep cohort study also determined that CT90% was a robust predictor of major adverse cardiovascular events (17). Nonetheless, few studies have evaluated the relationship between nocturnal hypoxemia and global CSVD burden. One study showed that ODI and minimum SpO_2_ were significantly correlated with burden of PVSs (38), while two other studies demonstrated that decreased arterial SpO_2_ and oxyhemoglobin saturation <90% were independently associated with more severe WMH load (39, 40). An animal model of CVSD demonstrated that OSA, simulated by intermittent tracheal balloon occlusion, could accelerate CSVD progression (41). Our findings reveal that the frequency and duration of oxygen desaturation, rather than the minimum SpO_2_, were associated with global CSVD burden covering a full range of individual MRI markers. Moreover, desaturation indices, including DesDur, can be extracted from limited channel sleep studies without electroencephalograms and are potentially more readily accessible.

The mechanisms linking OSA and CSVD may be multifactorial. OSA-related intermittent hypoxemia triggers endothelial dysfunction, vascular oxidative stress, systematic inflammation and glymphatic system dysfunction leading to brain dysfunction (42). A recent pilot neuroimaging study utilizing diffusion tensor imaging revealed that chronic hypoxic ischemia in the watershed region may contribute to the development of WMH (43), and another pilot study found both AHI and ODI were correlated with PVS burden (44). Given that OSA is a complex and heterogeneous disorder, and that AHI only measures the frequency of apnea and hypopneas and cannot provide information about the duration of respiratory events, indices such as DesDur and ODI incorporating the magnitude of hypoxemia might provide additional information. Although we cannot infer causality from our cross-sectional data, our findings have shown an association between oxygen desaturation and global CVSD burden, suggesting that nocturnal hypoxemia may contribute to the pathogenesis of CSVD.

NCPAP, the standard treatment for moderate-severe OSA, has been shown to improve white matter integrity (45) and microstructural changes in normal-appearing white matter (46) among patients with OSA, but failed to have long-term cardiovascular benefits among patients selected based on AHI only (47). A recent study in our sleep center found that sleep-related hypoxemia and mean heart rate on polysomnographic studies rather than AHI were determinants of incident major adverse cardiovascular events on longitudinal follow-up over a median of 8 years (17). Our results indicate that metrics quantifying nocturnal hypoxemia may be useful for selecting TIA/minor stroke patients at high risk of adverse effects of sleep apnea and thus may be useful to evaluate the effectiveness of CPAP treatment, as well as its impact on clinical outcomes relevant to CSVD, such as stroke recurrence and cognitive performance.

This study has some methodological limitations. First, this is a cross-sectional study of relatively small sample size involving predominantly Chinese. Our findings will need to be validated in larger prospective cohorts and randomized trials involving other ethnic groups to confirm the potential causal relationship and prognostic value of various indices of nocturnal hypoxemia in the development of CSVD, over and above conventional indices, such as AHI. Second, and largely due to the COVID-19 pandemic, only a small subset of the study population received HSAT, and hence, the results from the HSAT sub-study should be interpreted with caution. Moreover, HSAT cannot determine sleep stages. Third, we assumed that the CSVD burden remained largely the same during the one-year period after TIA/stroke onset. It is possible that a small proportion of individuals with uncontrolled vascular risk factors or severe OSA may have had CSVD progression during the follow-up period. Fourth, we used CSVD visual rating scales rather than more sensitive imaging markers to detect CSVD, such as WMH volume or cerebrovascular reactivity.

In this study, we demonstrated that in patients with TIA/minor stroke, OSA-risk measured by STOP-BANG and indices relating to the severity of nocturnal hypoxemia (ODI and DesDur) rather than AHI were significantly associated with a higher CSVD burden. Further large-scale prospective studies to determine whether OSA and related nocturnal hypoxemia may contribute to the pathogenesis of CSVD and whether these indices provide additional information on cerebral prognosis under nCPAP treatment, over and above AHI, are warranted.

## Data availability statement

The datasets presented in this article are not readily available because of ethical and privacy restrictions. Requests to access the anonymized datasets should be directed to KL, gkklau@hku.hk.

## Ethics statement

The studies involving human participants were reviewed and approved by Institutional Review Board of the University of Hong Kong/Hospital Authority Hong Kong West Cluster (IRB Reference No. UW18-361). The patients/participants provided their written informed consent to participate in this study.

## Author contributions

XL designed the study, collected data, did the statistical analysis and interpretation, wrote and revised the article. DL and MI provided study supervision and interpreted the data. KL conceived and designed the overall study, provided study supervision, interpreted data and revised the article. HM provided study supervision. All authors contributed to the article and approved the submitted version.

## Funding

We would like to thank the Hui Hoy and Chow Sin Lan Charity Fund Limited for their generous support in funding this study.

## Conflict of interest

The authors declare that the research was conducted in the absence of any commercial or financial relationships that could be construed as a potential conflict of interest.

## Publisher's note

All claims expressed in this article are solely those of the authors and do not necessarily represent those of their affiliated organizations, or those of the publisher, the editors and the reviewers. Any product that may be evaluated in this article, or claim that may be made by its manufacturer, is not guaranteed or endorsed by the publisher.
