# Supplementary material for: Associations of sleep apnea risk and oxygen desaturation indices with cerebral small vessel disease burden in patients with stroke
Source: Front Neurol. 2022 Aug 25;13:956208. doi: 10.3389/fneur.2022.956208 (PMC9452809; doi:10.3389/fneur.2022.956208)
Supplement: Supplementary file 1 [file Data_Sheet_1.docx]

Supplementary Material

*Association of sleep apnea risk and oxygen desaturation indices with cerebral small vessel disease burden in patients with stroke*

**List of Supplemental Methods, Tables and Figures:**

Nonstandard Abbreviations and Acronyms

Methods

Table I. Correlation of STOP–BANG score with MRI markers of CSVD

Table II. Baseline characteristics of patients who completed HSAT vs. patients who did not

Table III. Correlation of HSAT-derived indices with total CSVD score

Table IV. Association of AHI with individual MRI markers of CSVD (n=85)

Table V. Association of ODI with individual MRI markers of CSVD (n=85)

Table VI. Association of DesatDur with individual MRI markers of CSVD (n=85)

Figure I. Performance of STOP-BANG for diagnosis of AHI ≥5, ≥15 or ≥30/h

| **Nonstandard Abbreviations and Acronyms**  AHI apnea-hypopnea index  BG basal ganglia  CI confidence interval  CSO central semiovale  CSVD cerebral small vessel disease  CT90% percentage of total sleep time with an oxygen saturation <90%  CPAP continuous positive airway pressure  DesDur desaturation duration  HSAT home sleep apnea test  MRI magnetic resonance imaging  MTA medial temporal lobe atrophy  NIHSS National Institute of Health Stroke Scale  ODI oxygen desaturation index  OR odds ratio  OSA obstructive sleep apnea  PSG polysomnography  SpO_2_ oxygen desaturation  TOAST Trial of ORG 10172 in Acute Stroke Treatment  WMH white matter hyperintensities |
| --- |

**Methods**

**Definition of vascular risk factors**

**Hypertension**: resting systolic blood pressure ≥ 140 mmHg or a diastolic blood pressure ≥ 90 mmHg upon repeated measurements or treatment with anti-hypertensive medication.

**Diabetes mellitus**: fasting blood glucose level ≥ 7.0 mmol/L or prescription of oral hypoglycemic agents or insulin.

**Hyperlipidemia**: low-density lipoprotein-cholesterol level ≥ 4.1 mmol/L or total cholesterol ≥ 6.2 mmol/L.

**Alcohol drinking**: recent regular weekly alcohol consumption.

**Body mass index (BMI)**: estimated by dividing body weight by height squared (kg/m^2^).

**HSAT manual scoring method**

HSAT was manually scored by a qualified technician (MC) using the American Association of Sleep Medicine Scorning Manual Version 2.2 (1), blinded to the questionnaire and clinical data.

Apnea was scored when there was a drop in the peak signal excursion by $\geq$90% of baseline for $\geq$10 seconds using a nasal pressure cannula. Hypopnea was scored when there was $\geq$30% reduction in airflow for $\geq$10 seconds from baseline using nasal pressure in association with $\geq$3% arterial oxygen desaturation. Apnea-hypopnea index (AHI) was calculated as the average events of apneas and hypopneas per hour of sleep.

**Table I. Correlation of STOP–BANG score with MRI markers of CSVD (n=283)**

| **CSVD marker** | **Outcome variable** | **Spearman’s rho with STOP-Bang score** | ***p* value** |
| --- | --- | --- | --- |
| Global CSVD burden | Total CSVD score | 0.228 | **<0.001** |
| Lacune | Number of lacunes | 0.241 | **<0.001** |
| Microbleeds | Number of microbleeds | 0.085 | 0.157 |
| WMH | Periventricular Fazekas score | 0.112 | 0.061 |
|  | Deep Fazekas score | 0.136 | **0.022** |
|  | Total Fazekas score | 0.119 | **0.045** |
| PVS | PVS score in basal ganglia | 0.239 | **<0.001** |
|  | PVS score in central semiovale | 0.174 | **0.003** |
|  | Total PVS score | 0.241 | **<0.001** |
| Brain atrophy | Total brain atrophy score | 0.113 | 0.057 |
|  | MTA score | 0.139 | **0.019** |

CSVD = cerebral small vessel disease; MTA = medial temporal lobe atrophy; PVS = perivascular space; WMH = white matter hyperintensities

Total Fazekas score = Deep Fazekas score + Periventricular Fazekas score

Total PVS score = PVS score in basal ganglia + PVS score in central semiovale

**Table II. Baseline characteristics of patients who completed HSAT vs. patients who did not (n=283)**

|  | **All, n=283** | **No HSAT, n=198** | **HSAT, n=85** | ***p* value** |
| --- | --- | --- | --- | --- |
| **Baseline clinical characteristics** | | | | |
| Mean age, years | 65.2$\pm$12.0 | 66.3$\pm$12.3 | 62.7$\pm$11.0 | **0.038** |
| Male | 180 (64) | 124 (63) | 56 (66) | 0.602 |
| Hypertension | 158 (56) | 109 (55) | 49 (58) | 0.687 |
| Hyperlipidemia | 97 (34) | 64 (32) | 33 (39) | 0.291 |
| Diabetes | 81 (29) | 57 (29) | 24 (28) | 0.925 |
| Atrial fibrillation | 32 (11) | 25 (13) | 7 (8.2) | 0.285 |
| Prior TIA/stroke | 35 (12) | 28 (14) | 7 (8.2) | 0.167 |
| Ever smokers | 84 (30) | 63 (32) | 21 (25) | 0.230 |
| Alcohol drinker | 58 (20.6) | 40 (20.4) | 18 (20.9) | 0.920 |
| Systolic blood pressure, mmHg | 136$\pm$18 | 136$\pm$19 | 134$\pm$16 | 0.660 |
| Diastolic blood pressure, mmHg | 77 $\pm$13 | 76$\pm$14 | 78$\pm$11 | 0.113 |

Data are presented as mean $\pm$ standard deviation, or number (percentage)

HSAT = home sleep apnea test; TIA = transient ischemic attack

**Table III. Correlation of HSAT-derived indices with total CSVD score (n=85)**

| **PSG-derived indices** | **Spearman’s rho with total CSVD score** | ***p* value** |
| --- | --- | --- |
| AHI, events/hour | 0.212 | 0.052 |
| ODI, events/hour | 0.243 | **0.025** |
| DesDur, % | 0.217 | **0.046** |
| Minimum SpO_2_, % | -0.079 | 0.471 |
| CT90% | 0.223 | **0.040** |

AHI = apnea-hypopnea index, CSVD = cerebral small vessel disease, CT90% = percentage of total sleep time with an oxygen saturation<90%, DesDur = percentage of total desaturation time from total sleep time, HSAT = home sleep apnea test, ODI = oxygen desaturation index, SpO_2_ = oxygen desaturation;

**Table IV. Association of AHI with individual MRI markers of CSVD (n=85)**

| **CSVD markers** | **Outcome variables** | **Unadjusted OR (95% CI)** | **Multivariate-adjusted* OR (95% CI)** |
| --- | --- | --- | --- |
| Lacune | Presence of lacunes | 1.03 (1.00-1.06) | 1.01 (0.98-1.05) |
| Microbleeds | Presence of microbleeds | 1.01 (0.98-1.05) | 1.01 (0.98-1.05) |
| WMH | Periventricular Fazekas score | 1.02 (0.99-1.05) | 1.01 (0.98-1.05) |
|  | Deep Fazekas score | **1.03 (1.01-1.06)** | 1.04 (1.00-1.08) |
| PVS | Basal ganglia PVS score | **1.04 (1.01-1.07)** | **1.04 (1.01-1.07)** |
|  | Central semiovale PVS score | 1.02 (0.99-1.04) | 1.01 (0.98-1.04) |
| Brain atrophy | Total brain atrophy score quantile | 1.02 (0.99-1.06) | 1.03 (0.99-1.06) |
|  | MTA score | 1.01 (0.98-1.04) | 1.01 (0.98-1.04) |

AHI = apnea-hypopnea index; CI = confidence interval; CSVD = cerebral small vessel disease; MTA = medial temporal lobe atrophy; OR = odds ratio PVS = perivascular spaces; WMH = white matter hyperintensities;

Bold font indicates statistical significance with *p* value$<$0.05

^*^Adjusted for age, sex, vascular risk factors (hypertension, diabetes, atrial fibrillation, history of stroke/TIA, BMI, smoking), alcohol-use and total sleep time

**Table V. Association of ODI with individual MRI markers of CSVD (n=85)**

| **CSVD markers** | **Outcome variables** | **Unadjusted OR (95% CI)** | **Multivariate-adjusted* OR (95% CI)** |
| --- | --- | --- | --- |
| Lacune | Presence of lacunes | **1.03 (1.01-1.06)** | 1.02 (0.98-1.06) |
| Microbleeds | Presence of microbleeds | 1.02 (0.99-1.06) | 1.02 (0.98-1.06) |
| WMH | Periventricular Fazekas score | 1.02 (0.99-1.06) | 1.02 (0.98-1.05) |
|  | Deep Fazekas score | **1.03 (1.01-1.07)** | **1.04 (1.01-1.08)** |
| PVS | Basal ganglia PVS score | **1.04 (1.01-1.07)** | **1.04 (1.01-1.08)** |
|  | Central semiovale PVS score | 1.02 (0.99-1.05) | 1.01 (0.98-1.05) |
| Brain atrophy | Total brain atrophy score quantile | 1.02 (0.99-1.06) | 1.03 (0.99-1.07) |
|  | MTA score | 1.01 (0.98-1.05) | 1.01 (0.98-1.04) |

AHI = apnea-hypopnea index; CI = confidence interval; CSVD = cerebral small vessel disease; MTA = medial temporal lobe atrophy; ODI = oxygen desaturation index; OR = odds ratio; PVS = perivascular spaces; WMH = white matter hyperintensities.

Bold font indicates statistical significance with *p* value$<$0.05

^*^Adjusted for age, sex, vascular risk factors (hypertension, diabetes, atrial fibrillation, history of stroke/TIA, BMI, smoking), alcohol-use and total sleep time

**Table VI. Association of DesatDur with individual MRI markers of CSVD (n=85)**

| **CSVD markers** | **Outcome variables** | **Unadjusted OR (95% CI)** | **Multivariate-adjusted* OR (95% CI)** |
| --- | --- | --- | --- |
| Lacune | Presence of lacunes | **1.04 (1.01-1.08)** | 1.02 (0.98-1.07) |
| Microbleeds | Presence of microbleeds | 1.02 (0.98-1.07) | 1.02 (0.98-1.07) |
| WMH | Periventricular Fazekas score | 1.03 (0.99-1.07) | 1.02 (0.98-1.07) |
|  | Deep Fazekas score | **1.04 (1.01-1.08)** | 1.04 (1.00-1.09) |
| PVS | Basal ganglia PVS score | **1.05 (1.02-1.10)** | **1.05 (1.01-1.10)** |
|  | Central semiovale PVS score | 1.02 (0.98-1.06) | 1.01 (0.97-1.05) |
| Brain atrophy | Total brain atrophy score quantile | 1.03 (0.99-1.07) | 1.03 (0.99-1.08) |
|  | MTA score | 1.01 (0.98-1.05) | 1.01 (0.97-1.05) |

AHI = apnea-hypopnea index; CI = confidence interval; CSVD = cerebral small vessel disease; DesDur = percentage of total desaturation time from total sleep time; MTA = medial temporal lobe atrophy; ODI = oxygen desaturation index; OR = odds ratio; PVS = perivascular spaces; WMH = white matter hyperintensities

Bold font indicates statistical significance with *p* value$<$0.05

^*^Adjusted for age, sex, vascular risk factors (hypertension, diabetes, atrial fibrillation, history of stroke/TIA, BMI, smoking), alcohol-use and total sleep time

**Figure I. Performance of STOP-BANG for diagnosis of obstructive sleep apnea with AHI cut-off** $\boldsymbol{\geq}$**5,** $\boldsymbol{\geq}$**15 or** $\boldsymbol{\geq}$**30/h (n=85)**

a) AHI$\geq$5/h b) AHI$\geq$15/h

Area under ROC curve = 0.701 Area under ROC curve = 0.633


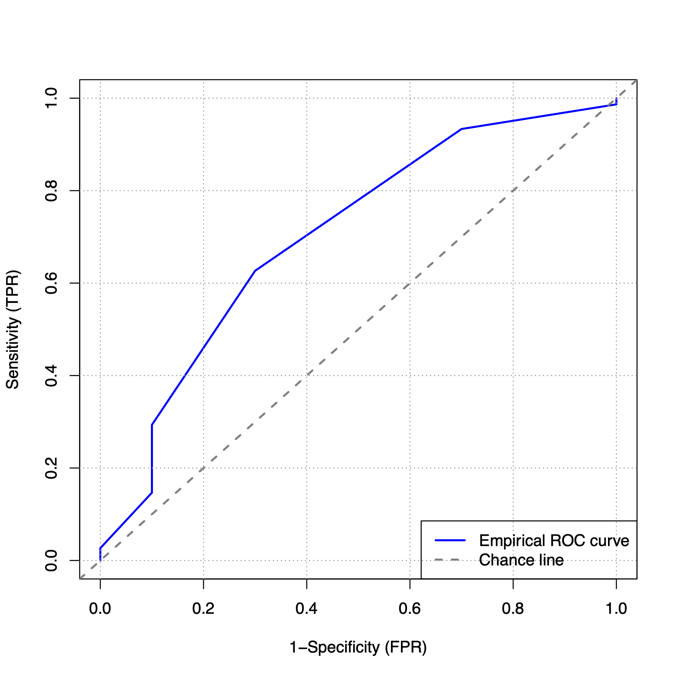

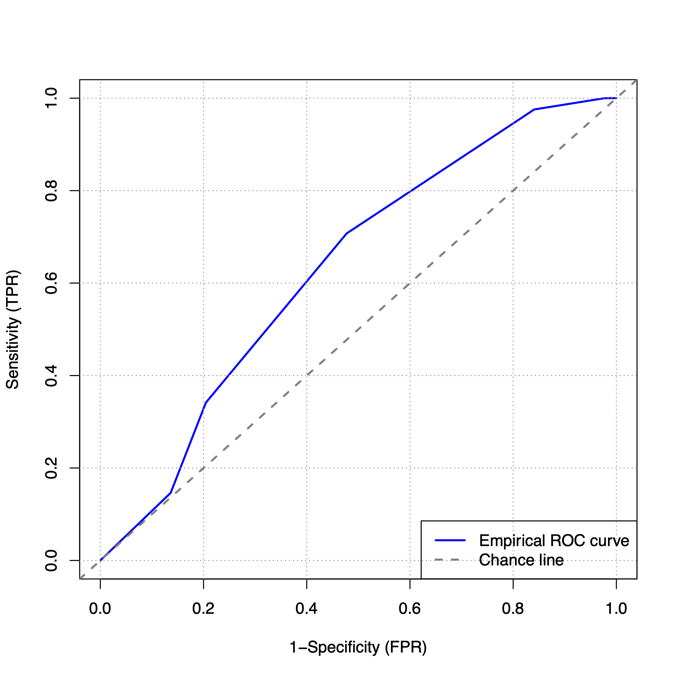


c) AHI$\geq$30/h

Area under ROC curve = 0.626


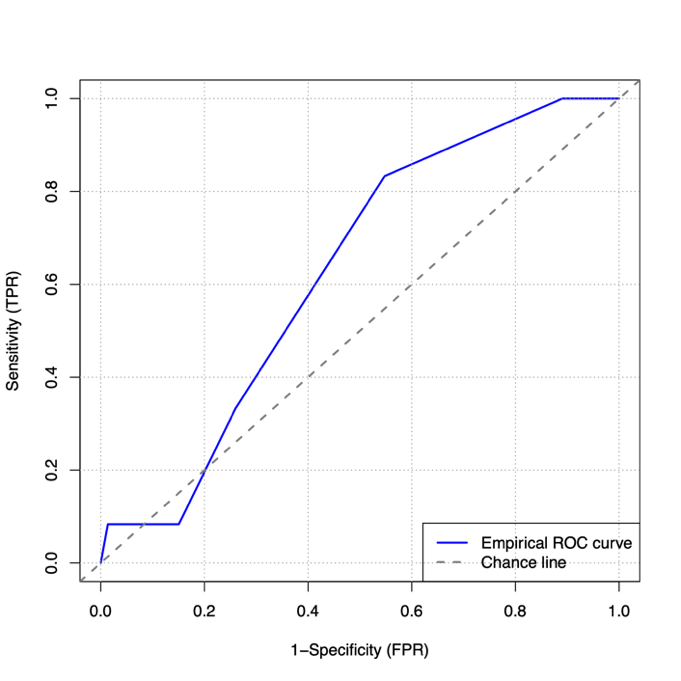


AHI = apnea-hypopnea index; ROC = receiver operating characteristic; TPR = true positive rate; FPR = false positive rate

**Reference:**

1. Berry RB, Gamaldo CE, Harding SM, Brooks R, Lloyd RM, Vaughn BV, Marcus CL. AASM Scoring Manual Version 2.2 Updates: New Chapters for Scoring Infant Sleep Staging and Home Sleep Apnea Testing. *J Clin Sleep Med* (2015) 11:1253–1254. doi: 10.5664/jcsm.5176
